# Transdiagnostic relevance of subjective cognitive complaints: a validation and population-based study using two Canadian scales (SSTICS and MoCA) in the UAE

**DOI:** 10.3389/fnbeh.2025.1677371

**Published:** 2025-11-12

**Authors:** Fadwa Al Mugaddam, Karim Abdel-Aziz, Syed Fahad Javaid, Javaid Nauman, Iffat ElBarazi, Emmanuel Stip

**Affiliations:** 1 Department of Psychiatry, College of Medicine and Health Sciences, United Arab Emirates University, Al-Ain, United Arab Emirates; 2 College of Medicine and Health Sciences, Public Health Institute, United Arab Emirates University, Al-Ain, United Arab Emirates; 3 Department of Psychiatry and Addictology, Faculty of Medicine, University of Montreal, Montreal, QC, Canada

**Keywords:** subjective cognitive complains, SSTICS, MOCA, cognition, schizophrenia, affective disorder

## Abstract

**Background:**

Cognitive disorders span several diagnostic categories in psychiatry, but subjective cognitive complaints (SCC) remain underutilized in transdiagnostic assessments, particularly in Arab contexts. These difficulties can also be present in Affective disorder illnesses are assessed using neuropsychological tests. Self-assessments are useful for understanding difficulties from the user’s perspective. The Subjective Scale to Investigate Cognition in Schizophrenia (SSTICS) is a rating scale designed to measure subjective cognitive complaints in persons with schizophrenia. This study explores the SSTIC-E, a culturally adapted tool, highlighting its cross-diagnostic relevance over simple psychometric validation.

**Methods:**

This cross-sectional study was conducted among 210 participants (126 patients, 84 controls) in the United Arab Emirates. Patients met ICD-10/DSM-5 criteria for schizophrenia spectrum disorders and affective disorders, in addition to other psychiatric disorders. The instruments included the SSTIC-E and the MoCA. Analysis focused on internal consistency, confirmatory factor analysis (CFA), and transdiagnostic comparisons.

**Results:**

Patients reported higher SSTIC-E scores than controls (mean = 34.06 vs. 22.55, *p* < 0.001). MoCA scores confirmed decreased objective performance in patients (mean = 22.71 vs. 27.19, *p* < 0.001). The SSTIC-E has excellent reliability (*α* = 0.89). No significant differences were observed in SCCs between the schizophrenia and affective disorder groups. CFA analysis confirmed a one-factor model with residual item correlations (CFI = 0.91, RMSEA = 0.058). Women reported higher SCC; age had no effect.

**Discussion:**

The SSTIC-E demonstrates utility beyond diagnostic silos, providing a valuable and culturally relevant instrument for transdiagnostic psychiatric assessment in Arabic-speaking populations. Schizophrenia exhibited slightly higher SCC compared to patients with affective disorders, with a lack of clear association between subjective and objective cognition. SCC is common across psychiatric diagnoses in the United Arab Emirates, supporting a dimensional model of cognitive dysfunction. SSTIC-E reveals insights into the lived experiences of patients not captured by objective tests. Cultural and gender influences underscore the necessity of context-specific approaches.

## Introduction

Mental health challenges are increasingly recognized in the Arab world, where factors such as conflict, displacement, social stigma, and limited access to care contribute to their high prevalence (Moselhy et al., 2012; El Khatib et al., 2023). In the United Arab Emirates (UAE), cultural factors further complicate mental health management (Elbarazi et al., 2025). Emotional expression is not widely encouraged within traditional cultural norms, which can lead to the suppression of feelings and make it difficult for individuals to seek or engage in therapy effectively (Al-Krenawi et al., 2009). Additionally, the UAE’s unique sociocultural context introduces specific challenges. Dialectical variations within the Arabic language can affect communication between therapists and patients, particularly when using standardized psychometric tools or therapeutic interventions, which may not be adapted to reflect regional linguistic nuances (Hamid and Darweesh, 2020). Moreover, psychometric research tools are often developed in English-speaking contexts, necessitating cultural and linguistic adaptation to ensure their relevance and effectiveness for diverse populations (Capitulo et al., 2001; Duffy, 2006).

Subjective cognition refers to an individual’s perceived cognitive abilities in daily life and plays a vital role in understanding mental states, which involves difficulties in concentration and thinking (Chelune et al., 1986; Derouesné et al., 1999). Evaluating subjective cognitive complaints is essential, as they provide insights into patients’ experiences that may not always align with objective cognitive measures.

Comparing subjective and objective cognitive measures is essential because these approaches provide complementary insight into an individual’s cognitive functioning.

Subjective measures, such as self-reported cognitive complaints, capture the patient’s perception of their subjective cognitive difficulties rather than their objective cognitive difficulties, which can often reflect their lived experience and emotional response to their condition (Lysaker et al., 2020; Allott et al., 2025).

However, they may be influenced by factors such as mood, anxiety, or self-awareness (Allott et al., 2025; Egeland et al., 2003). Objective measures, like standardized cognitive tests, offer an unbiased assessment of specific cognitive domains, ensuring that underlying impairments are accurately identified (Snyder et al., 2015).

Integrating both approaches is crucial for tailoring interventions, as subjective measures can guide clinicians in addressing patients’ perceived needs, while objective assessments provide a precise evaluation of cognitive deficits, leading to targeted treatments and rehabilitation strategies.

It is well established that patients with mental disorders like schizophrenia spectrum disorders present with cognitive deficits influencing their outcome. Neurocognitive disorders are a significant characteristic of the disease and are not simply the result of symptoms or deleterious effects of the treatment of schizophrenia.

Despite the clinical importance of the assessment of neurocognitive functioning in schizophrenia being well established, few instruments have been developed to respond to the cognitive complaints expressed by patients. A self-assessment of cognitive deficits in schizophrenia remains important in planning interventions related to functional outcomes and individual therapy (East-Richard et al., 2020).

The Subjective Scale to Investigate Cognition in Schizophrenia (SSTICS) offers a promising tool for addressing cognitive complaints in schizophrenia (Stip et al., 2003). This self-administered, 21-item scale evaluates specific cognitive domains affected by schizophrenia, such as memory and attention, and has been validated in multiple languages, including English (Stip et al., 2003), Italian, (Stratta et al., 2020), Spanish (Bengochea Seco et al., 2010), Mandarin (Chuang et al., 2019), Korean (Shin et al., 2016), Tunisian Arabic dialect (Johnson et al., 2009), Lebanese Arabic dialect (Haddad et al., 2021). As a first step, following research in translatology already described by Stip et al. (2024a), we aimed to validate the STICSS, as well as to evaluate the association of cognitive complaints with objective measures and other variables such as gender or diagnostic categories in the United Arab Emirates.

The so-called Subjective Scale to Investigate Cognition in Emirates (SSTIC-E) (Stip et al., 2024a), which is the adapted Emirati version of SSTICS, is particularly relevant for assessing subjective cognitive complaints in patients with schizophrenia in the UAE and the Arabian Gulf region (Stip et al., 2024a).

Cognitive dysfunction, a core feature of many psychiatric disorders, significantly affects clinical symptoms and functional outcomes (Allott et al., 2025; Lepage et al., 2014; Stip et al., 2017; Lecomte et al., 2007). While cognitive impairments are commonly associated with schizophrenia, they are also observed in conditions such as depression, dementia, and addiction (Abramovitch et al., 2021; Cotter et al., 2018). Furthermore, in the current context of transdiagnostic approaches, we wanted to explore whether the cognitive complaints expressed are measurable with our scale (Sheffield et al., 2017).

Several meta-analyses have been published to evaluate the impact of psychiatric disorders on neuropsychological functioning (East-Richard et al., 2020). Studies supporting the nonspecific nature of the association between cognitive dysfunction and psychopathology appear in cross-sectional and longitudinal studies (Abramovitch et al., 2021; Cotter et al., 2018; Chavez-Baldini et al., 2023). To our knowledge, this is the first study to compare, for instance, the cognitive complaints between schizophrenia spectrum and the affective and anxiety disorder spectrum, such as bipolar affective disorder (BAD), major depressive disorder (MDD), and generalized anxiety disorder (GAD), Mixed anxiety and depressive disorder.

## Method

### Study design and objectives

This research study is a cross-sectional validation study. This research’s main objectives are to study the relationship between subjective cognitive complaints (SSTIC-E) among clinical and healthy control subjects and the objective cognitive status, assessed using an objective Canadian tool that measures cognitive problems (the Montreal Cognitive Assessment Scale - MoCA) (Nasreddine et al., 2005). To explore the use of the two scales in a transdiagnostic approach. To our knowledge, this is the first time that a study has attempted to explore this latter objective.

The project was approved by the United Arab Emirates University, Social Sciences Ethical Committee (ERS_2021_8418), SEHA Research Ethics Committee (SEHA-IRB-040), and Tawam Hospital (No.: KD/AJ/849). The setting for clinical subjects was two tertiary hospitals, Tawam Hospital outpatient clinic (polyclinic) & Al Ain Hospital outpatient clinic (polyclinic), both hospitals located in the Al Ain district of Abu Dhabi Emirate.

The setting for healthy control subjects was community-based.

### From SSTICS to SSTIC-E

The scale consists of five domains to assess subjective complaints in the form of a Likert-type self-assessment questionnaire, including a total of 21 questions, where the person needs to choose as follows (4 - very often; 3 - often; 2 - sometimes; 1- rarely; 0 - never). The scale consists of five domains to assess subjective complaints, the first domain assesses memory in two forms: working memory in questions 1 & 2, and explicit long working memory (episodic memory, questions 3–9, and semantic memory in questions 10 & 11) (Stip et al., 2003).

The second domain assesses attention in five sub-domains from question 12 to question 16; (distractibility Q12, alertness Q13, selective attention Q14, divided attention Q15, and sustained attention Q16) (Stip et al., 2003).

The third domain evaluates executive functions in questions 17, 18, and 19, by asking about planning in Q17, organization in Q18, and flexibility in Q19. The fourth domain for language assessment in question 20 and question 21, which is the last on the scale, is for praxis assessment, the internal scale consistency (*α* = 0.86) (Stip et al., 2003).

The initial translation aims to develop the first version of the tool in the language used in the UAE. A native speaker in both languages, Arabic and English, performed the initial translation. The process of adapting the subjective scale to investigate cognition in schizophrenia (SSTICS) to produce the subjective scale to investigate cognition in the Emirate population (SSTIC-E) was performed through four main stages and described in a comprehensive previous article. The process of translation and cultural adaptation of the SSTICS to produce the Subjective Scale of Cognitive Investigation in the Emirati Population (SSTIC-E) took place in four main stages and has been extensively described in a previous detailed article (Stip et al., 2024a).

### Using MoCA with SSTIC-E

MoCA is a widely used cognitive screening tool that assesses various cognitive domains, including attention, memory, language, and executive function. It has been extensively validated in both clinical and research settings and has demonstrated high sensitivity and specificity for detecting mild cognitive impairment (Amro et al., 2025; Li et al., 2023).

In the UAE, the MoCA has been primarily validated for detecting cognitive impairment and dementia; however, recent local studies have also utilized MoCA to explore cognitive performance among psychiatric populations, such as depression and schizophrenia, demonstrating its cross-diagnostic applicability.

Although the two tools SSTICE-E and MoCA assess various aspects of cognition, they can be used together to provide a more comprehensive assessment of cognitive function in individuals with schizophrenia.

In summary, the MoCA and SSTICS tools can be used together to provide a more comprehensive assessment of cognitive function in individuals with schizophrenia, and using both tools can help identify discrepancies between objective and subjective measures of cognitive function. Both MoCA and SSTICS tools assess similar cognitive domains. These domains include memory, orientation, attention, language, visuospatial abilities, and executive functions. However, there may be differences in the specific subtests used to assess these domains between the two tools.

### Methods of selecting participants

Clinical participants were recruited from the outpatient psychiatry departments of Tawam and Al Ain Hospitals, where patients receive pharmacological and psychotherapeutic interventions per ministry of health (SEHA) clinical guidelines. The control group was drawn from community members in Al Ain, including volunteers, workers, university staff, students without psychiatric history, confirmed through self-report and clinical screening.

The study utilized snowball sampling techniques to recruit participants. Eligibility was determined based on the following criteria:

Participants were selected based on specific inclusion and exclusion criteria to ensure the study’s relevance and reliability. For the clinical group, inclusion criteria required adult male and female patients aged 18 years and above, fluent in Arabic, and willing to participate in the study as well as diagnoses with Schizophrenia Spectrum disorder, an affective or anxiety disorder (BAD, MDD, GAD, OCD, Panic Disorder, Mixed anxiety and depression disorder, adjustment disorder) or a personality disorder confirmed by senior psychiatrists using DSM-5 or ICD-10 criteria, based on structured clinical interviews.

While exclusion criteria for patients included individuals who were below 18 years of age or unwilling to take part in the study, most importantly, patients with cognitive impairments, such as a diagnosis of intellectual disabilities, substance abuse, major neurological disorders, or intellectual disability, were excluded from the study. For the control group, inclusion criteria included healthy individuals without mental disorders, Arabic-speaking adult males, and females aged 18 years or older who agreed to participate, while exclusion criteria ruled out those under 18 or individuals unwilling to participate.

To the best of our knowledge, this is the first formal study conducted in the UAE to use the MoCA for the objective assessment of cognitive impairment in people with mental disorders. While several recent regional initiatives have used the MoCA to examine the cognitive profiles of patients with depression and schizophrenia, these have generally been conducted on a smaller scale. Thus, as in other countries, the MoCA is used by psychiatric clinicians and residents in clinics and hospitals. In the UAE, the MoCA has primarily been validated for the detection of cognitive impairment and dementia; however, emerging local research has also applied it to psychiatric populations, including people with depression and schizophrenia.

### Sample size

To validate SSTIC-E, *a priori* sample size target was set to ensure adequate power for scale validation. With 210 respondents for a 21-item instrument, our sample meets common psychometric recommendations of ≈10 participants per item and exceeds widely used absolute minimums (100–200) for exploratory and confirmatory factor analysis.

### Data collection

Participants who agreed to take part in the study, following their consent, were requested to complete a socio-demographic questionnaire, which served as the primary data collection instrument. Additionally, they underwent measurements, including the administration of the Subjective Scale for Investigating Cognition-Emirates (SSTIC-E) for subjective assessments, and the MoCA.

The researcher was trained and certified by completing the full MoCA training (CERTIFIED RATER- ID AEALMDA 71056214301). The MoCA has 30 questions and can be finished in 10–12 min. Although it is a helpful screening test, the results of other tests must also be considered to confirm a diagnosis.

The test evaluates six domains: the Visuospatial/executive domain, Naming, Memory, Attention, Language, Abstraction, delayed recall, and Orientation. The scale scores the subject to have a normal cognitive function if the subject scores 26 /30 (Nasreddine et al., 2005). Furthermore, to enhance data accuracy and comprehensiveness, we cross-validated certain participant-provided information by referencing electronic medical records (EMR) at SEHA, through a system named SLAMTAK. We entered the data in the SPSS program for descriptive analysis and used R software version 4.4.1 and the lavaan package for confirmatory analyses (Lau et al., 2021).

The study did not include standardized measures of psychopathology severity (e.g., PANSS, HDRS), which limits our ability to correlate symptom intensity with subjective cognitive complaints. Future studies should include such assessments to better elucidate these relationships.

## Results

The Arabic version of the SSTIC-E was administered to a total of 210 participants, including 126 patients and 84 healthy control participants. Table 1 presents the typical characteristics of participants, including demographic information such as age, gender, marital status, nationality, residency emirate, educational status, and employment status. The table also provides the number and percentage of participants in each category and the mean, minimum, and maximum age.

**Table 1:** Participants general characteristics.

Variables *n* (%)	Participants 210 (%)	Control 84 (40%)	Patients 126 (60%)
Age	(Mean ± SD)	(36.07 ± 11.61)	(40.36 ± 15.28)
	Age range	(18–73) years	(18–82) years
Gender	Male	33 (26.2%)	54 (42.9%)
	Females	51 (40.5%)	72 (57.1%)
Marital status	Married	40 (31.7%)	76 (60.3%)
	Single	41 (32.5%)	40 (31.7%)
	Divorced	1 (0.8%)	7 (5.6%)
	Widowed	1 (0.8%)	3 (2.4%)
	Engaged	1 (0.8%)	0
Nationality	Emirati	39 (31.0%)	89 (70.6%)
	Non-Emirati	45 (35.7%)	37 (29.4%)
Residency emirate	Abu Dhabi	82 (65.1%)	121 (96.0%)
	Dubai	0	1 (0.8%)
	Sharjah	2 (1.6%)	0
	Ajman	0	2 (1.6%)
	Fujairah	0	1 (0.8%)
	Ras Al Khaima	0	1 (0.8%)
	Umm Al Quwain	0	0
Educational status	No Formal Education	1 (0.8%)	4 (3.2%)
	Primary School Level	3 (2.4%)	11 (8.7%)
	Preparatory School Level	0	14 (11.1%)
	Secondary School Level	17 (13.5%)	43 (34.1%)
	University Level	39 (31.0%)	50 (39.7%)
	Higher Education	24 (19.0%)	4 (3.2%)
Employment status	Employee (Full time)	52 (41.3%)	26 (20.6%)
	Employee (Part-time)	7 (5.6%)	8 (6.3%)
	Studying	13 (10.3%)	8 (6.3%)
	Not working	6 (4.8%)	53 (42.1%)
	Retired	1 (0.8%)	11 (8.7%)
	Housewife	5 (4%)	20 (15.9%)

In terms of age, the participants had a mean age of 36.07 years, with a range of 18 to 73 years. The gender distribution was 33 males (39.3%), and the rest were 51 (60.7%) in the control group, 54 males (42.9%) and the rest is 72 (57.1%) in the patient group. Regarding the recruitment sites of control (non-psychiatric patients) settings, 47 participants (37.3%) stayed in the family or parental settings, while the remaining participants were divided among several other locations (see Table 1).

Regarding educational status, most participants had completed at least secondary school (31.0% at the university level and 19.0% with higher education).

As for employment status, most of the participants were employees (full-time or part-time) or students, while a smaller number were not working, retired, or housewives Table 1 depicts the demographic characteristics of the study participants.

### Participants clinical features

In the control group (non-psychiatric patients), 4.8% of participants reported having diabetes, while 61.9% did not have diabetes. In the patient group, 25.4% of participants reported having diabetes, while 74.6% did not. Similarly, in the patient group, 14.3% of participants reported having major depressive disorder (MDD), while in the control group, no participants reported having this disorder (see Table 2).

**Table 2:** Participants clinical features.

*n* (%)	Control 84 (40%)		Patients 126 (60%)		CI 95%	*p* value
Disease	Yes (%)	No (%)	Yes (%)	No (%)		
Diabetes Mellitus	6 (4.8%)	78 (61.9%)	32 (25.4%)	94 (74.6%)	(0.132, 0.361)	0.0002
Hypertension	5 (4.0%)	79 (62.7%)	19 (15.1%)	107 (84.9%)	(0.018, 0.246)	0.025
Cardiovascular Diseases	5 (4.0%)		18 (14.3%)	108 (85.7%)	(0.020, 0.246)	0.044
Neurological Diseases			3 (2.4%)	123 (97.6%)		
Chronic Obesity			2 (1.6%)			
Insomnia			4 (3.2%)			
Psychiatric Diseases			124 (98.4%)	2 (1.6%)		
Adjustment Disorder			3 (2.4%)			
Affective Bipolar Disorder			6 (4.6%)			
Bipolar Disorder Order			21 (16.6%)			
Personality Disorder			2 (1.6%)			
General Anxiety Disorder			15 (11.9%)			
Major Depression Disorder			22 (14.3%)			
Mixed Anxiety and Depression Disorder			12 (9.5%)			
Obsessive Compulsive Disorder			4 (3.2%)			
Schizophrenia			30 (23.8%)			
Panic Attack			5 (3.9%)			

DM, Diabetes Mellitus; Htn, Hypertension; CVD, Cardiovascular; ND, Neurological Diseases; CO, Chronic Obesity; PD, Psychiatric Diseases; AD, Adjustment Disorder; ABD, Affective Bipolar Disorder; BD, Bipolar Disorder; PD, Personality Disorder; GAD, General Anxiety Disorder; MDD, Major Depression Disorder; MADD, Mixed Anxiety and Depression Disorder; OCD, Obsessive Compulsive Disorder; SCZ, Schizophrenia; PA, Panic Attack.

All patients had psychiatric disorders, with schizophrenia (24.2%), bipolar affective disorder (BAD) (21.8%), major depressive disorder (MDD) (17.7%), and general anxiety disorder (GAD) (15.1%), Mixed anxiety and depressive disorder (9.6%) being the most common diagnoses. Other diagnoses were reported in smaller percentages. To explore differences between schizophrenia and affective disorder, two groups of patients were formed from the total sample; the first linked to schizophrenia spectrum diagnoses, and the second linked to the spectrum diagnosis of affective illnesses (see Table 2).

## Participants’ SSTIC-E and MOCA scores

### Comparison between groups on the SSTIC-E and MOCA scores

The comparison was realized using a t-test with Welch adjustment (for unequal variance), and the standard deviation (SD).

**Figure 1:**
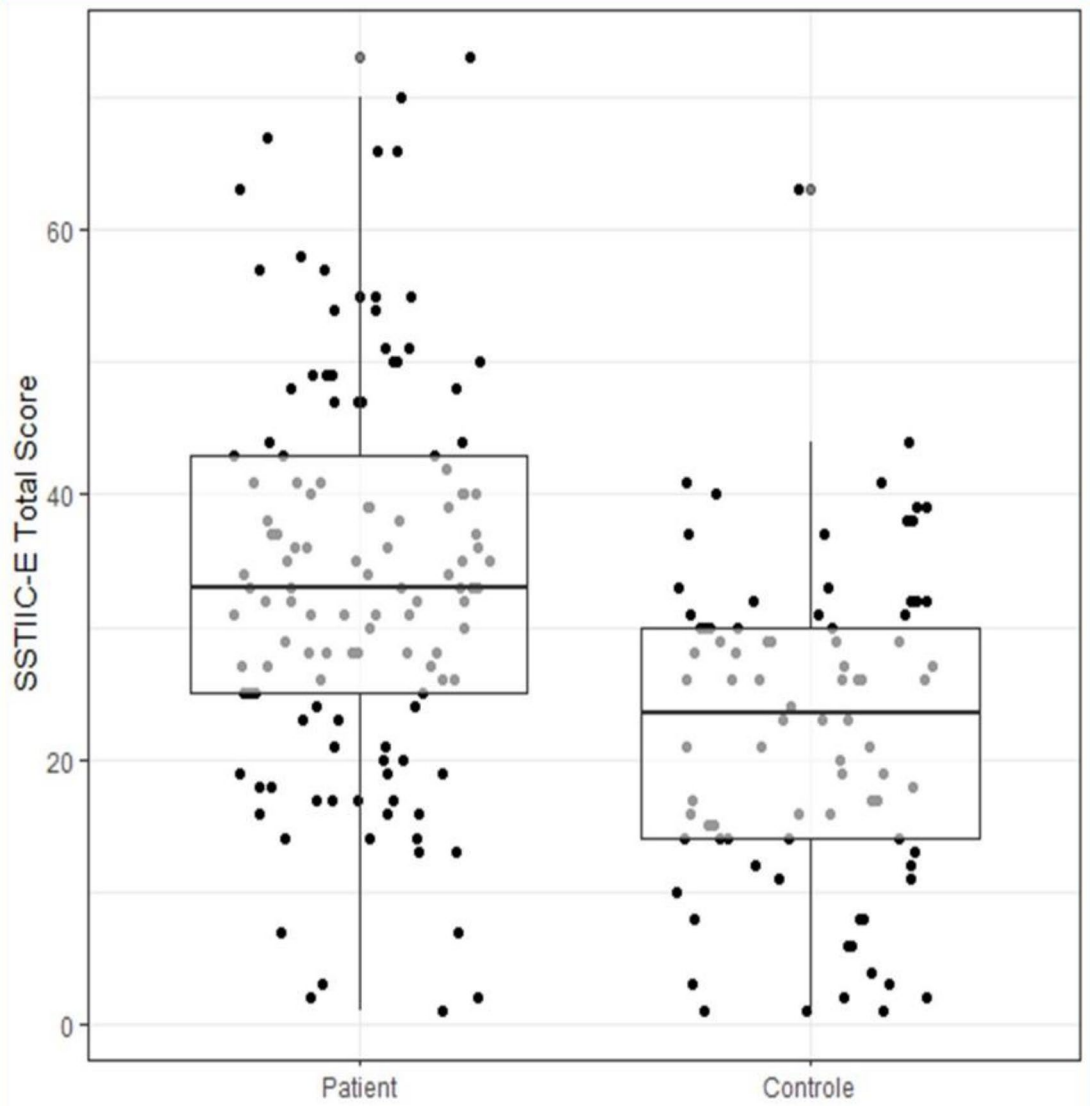
Comparison of SSTIC-E scores between groups.

The SSTIC-E score was much higher on average in patients (34.06, SD = 15.19) than in controls (22.55, SD = 12.04), (95% CI: −13.51, −7.87), (see Figure 1), and the difference is statistically significant (t_202 = 6.17, *p* < 0.001).

**Figure 2:**
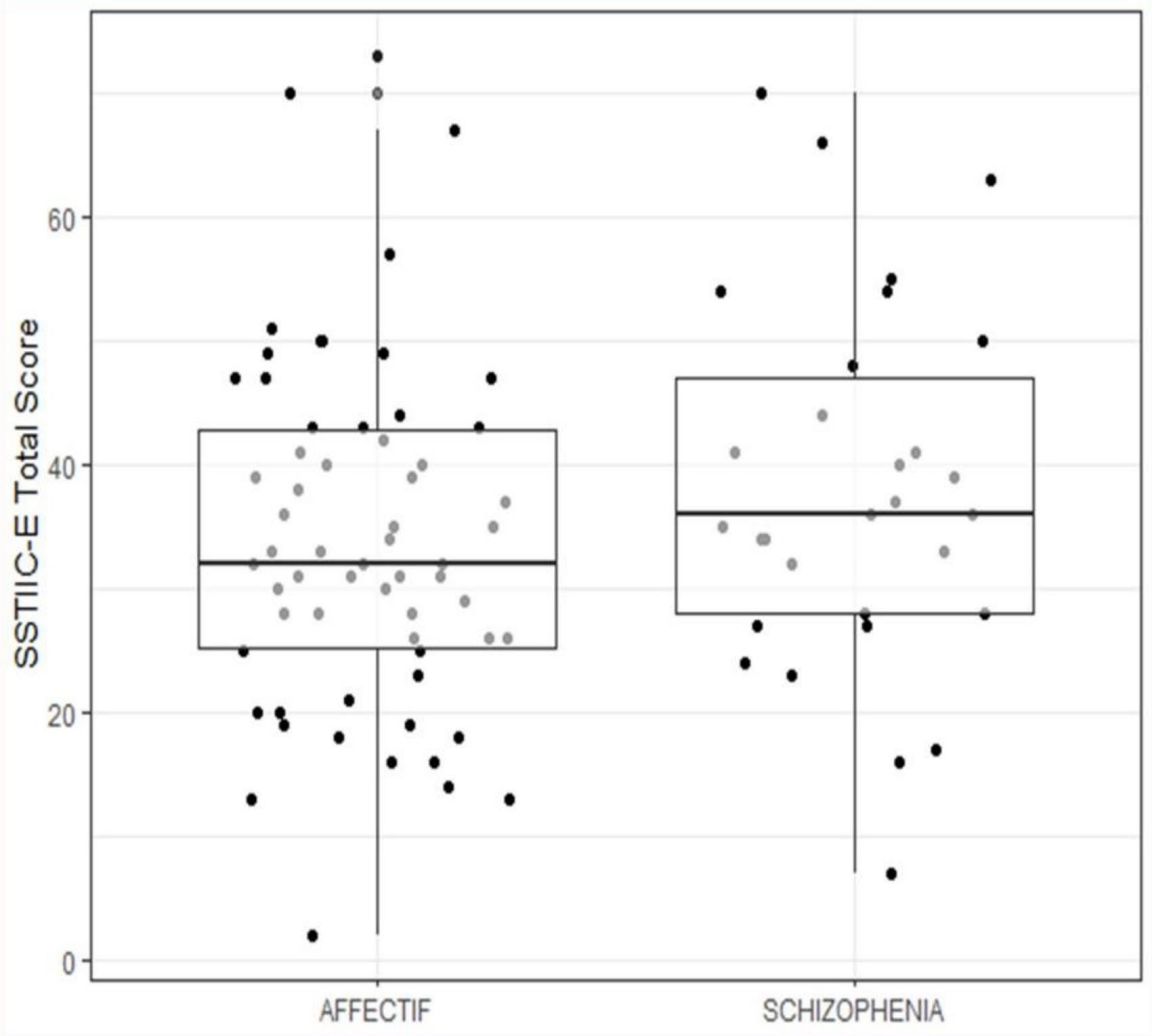
SSTIC-E score by group.

**Figure 3:**
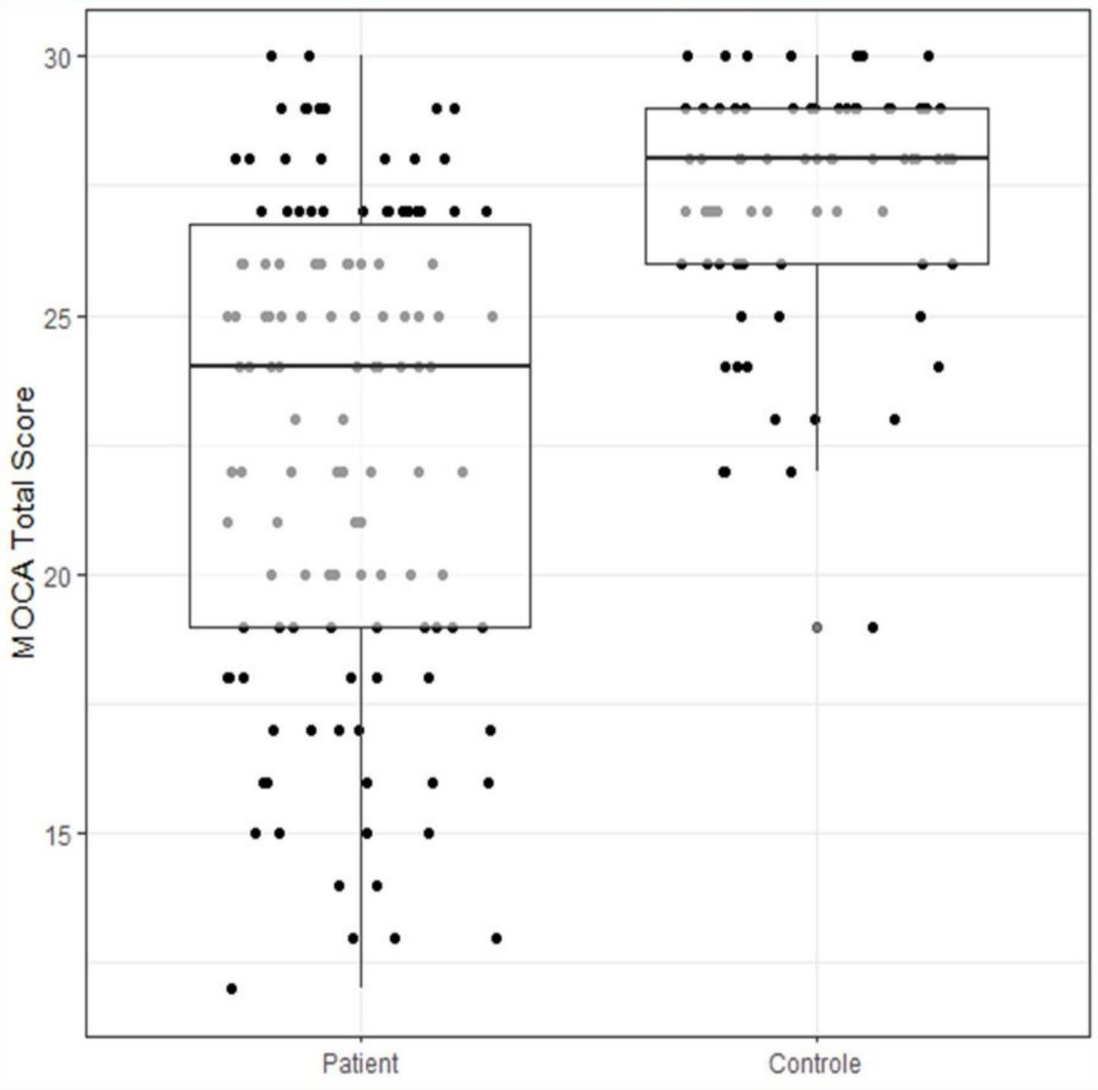
Comparison of MoCA scores between groups.

The MOCA score (Figure 2) is much lower on average in patients (22.71, SD = 4.58) than in controls (27.19, SD = 2.24), (95% CI: 3.08, 5.87), (see Figure 3), and the difference is statistically significant (t_193 = −9.41, *p* < 0.001).

### Confirmatory factor analysis on the structure of the SSTICS

**Figure 4:**
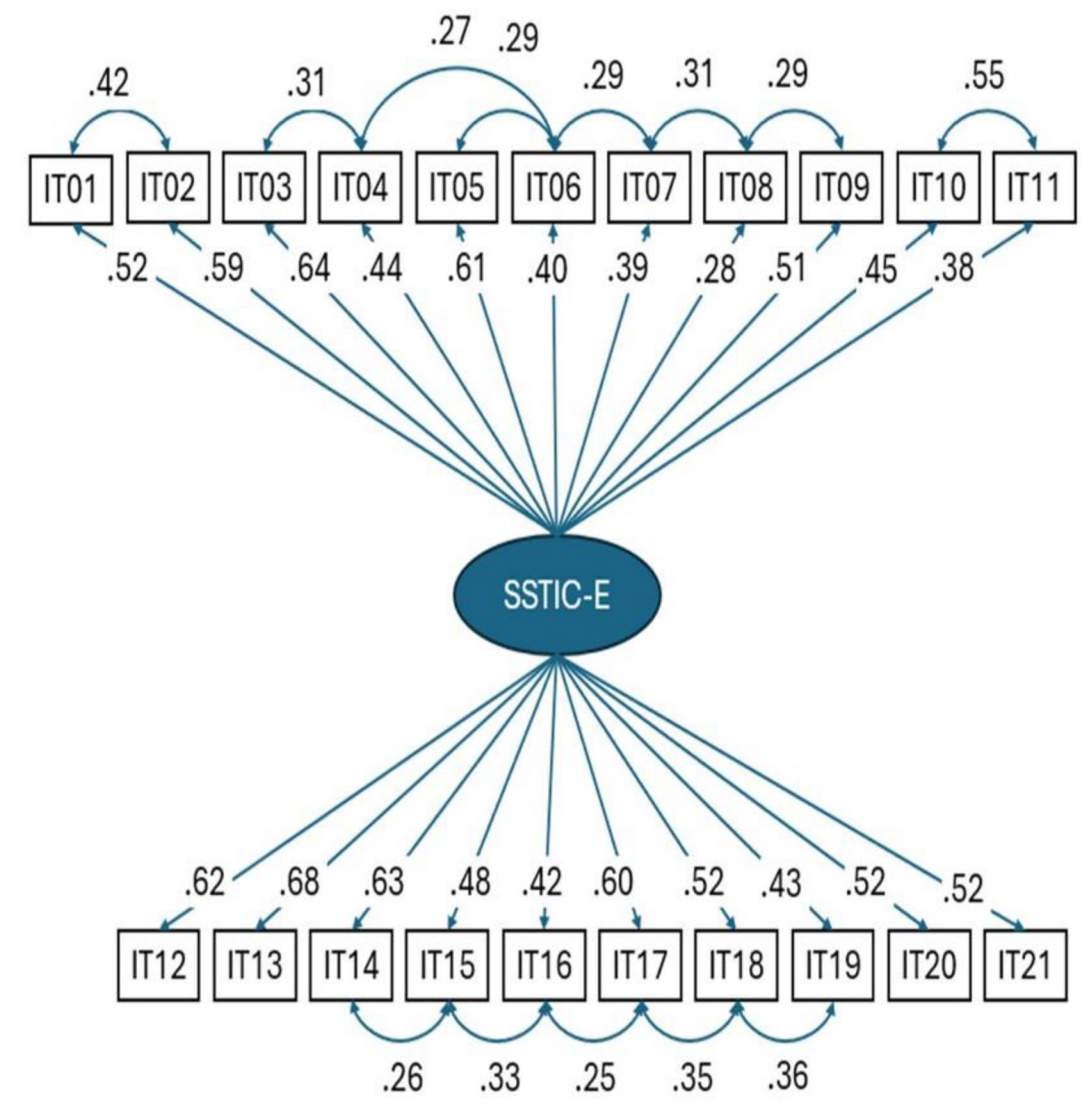
Factorial analysis on SSTICS-E.

A confirmatory factor analysis was conducted on the items of the SSTICS-E. The study was conducted on patients only. For the 1-factor raw model, the fit indices are not satisfactory (CFI = 0.683, RMSEA = 0.11, and SRMR = 0.09). Hu and Bentler recommend CFI ≥ 0.90, RMSEA < 0.6, and SRMR < 0.08. These indices are, however, improved by considering the residual correlations between certain items as illustrated in Figure 4. The new indices conform to a good model (CFI = 0.91, RMSEA = 0.058, SRMR = 0.072).

Cronbach’s alpha coefficient was 0.89 (95% CI: 0.86–0.91), which shows that the instrument has excellent reliability. The residual correlations shown represent that some items share a common variance beyond the common factor. For example, items 1 and 2 deal with memory, and items 18 and 19 deal with task organization and remain correlated beyond the general factor (see Figure 4).

### Effect of age and gender on the SSTICS-E

We evaluated whether there are age or gender effects on the SSTICS-E. As there was a group effect, we added age and gender in linear regression (see Table 3).

**Table 3:** Effect of age and gender on the SSTICS-E.

Effect	Est.	É.-T.	t	P(>|t|)	Inf. 95% CI	Supp. 95% CI
(Intercept)	38.95	3.21	12.15	<0.001	32.63	45.27
Age (in years)	−0.08	0.07	−1.11	0.27	−0.21	0.06
Male	−4.18	1.95	−2.14	0.033	−8.03	−0.34
Control	−12.11	1.98	−6.11	<0.001	−16.01	−8.20

We can see that age in years has no impact on the SSTICS-E, but that men are 4.18 points lower than women (*p*-value <0.001) (see Table 3).

### Transdiagnosis approach: comparison of groups on SSTIC-E score

The SSTIC-E score is slightly higher in patients suffering from schizophrenia (m = 38.0) as opposed to patients suffering from affective disorders (BAD, MDD) (m = 34.0); however, the difference is not statistically significant (t55 = −1.24, *p* = 0.22) (see Figure 2).

## Discussion

In this study, patients with schizophrenia exhibited slightly higher subjective cognitive complaint scores on the SSTIC-E (*m* = 38.0) compared to patients with affective disorders (bipolar and depression) (*m* = 34.0). However, this difference was insignificant (t55 = −1.24, *p* = 0.22). This finding suggests that while subjective cognitive complaints may be more pronounced in schizophrenia than in affective disorders, the overlap between the two conditions is substantial, which aligns with the transdiagnostic nature of cognitive impairments in psychiatric disorders (Egeland et al., 2003). Several potential reasons may account for this finding.

First, the overlap in cognitive impairments between schizophrenia and affective disorders could contribute to the lack of a significant difference. Both conditions are characterized by deficits in memory, attention, executive functioning, and other cognitive domains, underscoring their transdiagnostic nature. The subjective cognitive complaints reported by participants may be reflective of shared underlying cognitive dysfunctions, rather than differences in the severity or type of impairment between the two groups.

Second, the subjective nature of the SSTIC-E scores may have influenced the results.

Self-reported cognitive complaints can be shaped by individual self-awareness of their cognitive deficits, illness insight, and mood states, which might differ between individuals with schizophrenia and those with affective disorders. For instance, individuals with affective disorders may overreport cognitive issues due to depressive symptoms, while those with schizophrenia may underreport them due to reduced illness insight. Third, the sample size may have limited the power to detect statistically significant differences. A larger sample size might provide greater precision and allow for the detection of subtle differences in subjective cognitive complaints between these two groups. Lastly, the clinical heterogeneity within each group could have played a role. Both schizophrenia and affective disorders encompass a spectrum of severity and symptomatology, which could obscure distinctions in subjective cognitive complaints when assessed at the group level.

These findings emphasize the importance of considering the complex interplay of overlapping cognitive problems, individual differences in reporting, and methodological limitations when interpreting subjective cognitive assessments in psychiatric populations.

Future research with larger, more homogenous samples and multimodal assessment approaches (e.g., combining subjective and objective measures) is needed to better delineate the nuances of cognitive impairments across psychiatric conditions.

### Comparison to literature

The study revealed that patients (*n* = 126) had significantly higher subjective cognitive complaints on the SSTIC-E compared to the control group (*n* = 84). Patients also outscored controls across all cognitive domains, including memory (17.58 vs. 13.50), attention (5.81 vs. 3.65), executive functions (3.01 vs. 2.52), language (0.90 vs. 0.60), and praxia (0.24 vs. 0.13).

These findings suggest a consistent difference between clinical and non-clinical populations. Compared with previous literature, the current study’s patient scores were higher than those reported by Stip et al. (2003) (25.94, SD = ±9.72) and Raffard et al. (2020) (25.56, SD = ±9.10), though control scores aligned closely (22.55 vs. 22.55). Other studies, such as Stratta et al. (2020) in Italy (mean = 23.34), Lecardeur et al. (2009) in France (mean = 24.73), and Haddad et al. (2021) in Lebanon (mean = 25.15) showed comparable patient scores. In contrast, studies from Asia, including Baliga et al. in India (mean = 16.22), Shin et al. (2016) in Korea (mean = 18.9), and Chuang et al. (2019) in Taiwan (mean = 16.22), reported lower mean SSTIC scores, highlighting possible cultural or clinical differences.

In comparing the findings from the literature with our study results, several key patterns and discrepancies emerge regarding subjective cognitive complaints in schizophrenia patients, as well as the role of scales like SSTICS in assessing these complaints in other medical conditions (Stip et al., 2024b).

Recent literature supports the observation that subjective cognitive complaints are prevalent in both schizophrenia and affective disorders, but they may manifest differently. For instance, cognitive deficits in schizophrenia are often considered more severe and pervasive, impacting domains such as working memory, attention, and executive functioning (Barch et al., 2022). By contrast, patients with affective disorders, such as bipolar disorder and major depressive disorder, often report subjective cognitive difficulties linked to episodic memory, attention, and mental processing speed (Gualtieri and Johnson, 2008; Bora and Pantelis, 2016).

In terms of subjective cognitive complaints measured by tools like the SSTIC-E, studies have reported that schizophrenia patients rate their cognitive issues as more disabling compared to patients with affective disorders (Moritz et al., 2014).

This aligns with the findings that schizophrenia involves a more profound disruption in self-monitoring and metacognition, which can amplify the perception of cognitive dysfunction (Allott et al., 2025). However, the non-significant difference observed in this study may reflect individual variability in the subjective appraisal of cognitive impairments or the specific characteristics of the patient sample.

### Reliability and consistency of SSTICS

Studies such as those by Stip et al. (2003) and Johnson et al. (2009) report strong internal consistency and reliability of the SSTICS scale, demonstrating its effectiveness for assessing subjective cognitive complaints in schizophrenia. Additionally, Lecardeur et al. (2009) confirmed the convergent validity of SSTICS with other cognitive measures, reinforcing its utility in schizophrenia assessments.

Similarly, our study validated the Arabic version of the SSTIC-E, achieving high internal consistency with a Cronbach’s alpha coefficient of 0.89 (95% CI: 0.86–0.91). However, the confirmatory factor analysis indicated that while the 1-factor model did not fit well, a revised model with additional item correlations (e.g., memory and task organization) improved fit indices (CFI = 0.91, RMSEA = 0.058, SRMR = 0.072). This suggests that, like previous studies, SSTICS is a reliable tool, but there might be a need to account for item-specific correlations.

Many studies, including Prouteau et al. (2004) and Potvin et al. (2005), indicate weak correlations between subjective cognitive complaints and objective cognitive complaints measures in schizophrenia. Potvin et al. (2014) highlighted that schizophrenia patients tend to over-report cognitive difficulties, even when objective measures do not show corresponding deficits.

Our study mirrored these findings, showing that the patient group had significantly higher SSTIC-E scores compared to controls (mean = 34.06 vs. 22.43), and these differences were statistically significant. However, as seen in the confirmatory factor analysis, some residual correlations between items suggest that subjective complaints could reflect additional factors beyond general cognitive impairment, like what Potvin et al. (2014) proposed about subjective cognition being influenced by factors like self-perception.

### Demographic variables (age and gender)

Gender and age effects on cognitive complaints have been explored in several studies. Stip et al. (2022) found some gender differences in cognitive complaints, with women reporting more issues than men, although this was not statistically significant. Other studies have found mixed results regarding age and cognitive complaints. In our study, we observed that while age had no impact on the SSTIC-E scores, gender differences were significant, with males scoring 4.8 points lower than females. This finding aligns with the literature suggesting some gender-related variation in cognitive complaints, but it highlights the need for further research to fully understand these dynamics.

In many cultural contexts, including those in the UAE and similar regions, gender roles and societal expectations often shape how people perceive, experience, and express health-related concerns.

Women may be more attuned to or comfortable acknowledging and reporting cognitive difficulties due to social norms encouraging emotional expression and seeking help.

In contrast, men may underreport such complaints due to stigma, societal pressures to appear resilient, or a reluctance to acknowledge perceived vulnerabilities, including cognitive challenges.

Additionally, cultural factors may influence how cognitive difficulties are experienced and reported. For instance, women might face greater cognitive demands in managing household responsibilities, caregiving roles, or multitasking, which could heighten their awareness of cognitive changes or difficulties. On the other hand, men might attribute such issues to external factors (e.g., stress or fatigue) rather than internalizing them as cognitive problems, resulting in lower scores on subjective cognitive assessments like the SSTIC-E.

Understanding these cultural and social influences is crucial for interpreting gender differences in cognitive complaints and for tailoring interventions to address the unique needs and reporting tendencies of men and women. Further research into how cultural norms and societal expectations shape the perception and reporting of cognitive difficulties can provide valuable insights for improving assessment and care strategies.

We also applied a transdiagnostic approach, comparing patients with schizophrenia and affective disorders. Interestingly, while the SSTIC-E score was higher in schizophrenia patients (mean = 38.0), the difference compared to patients with affective disorders was not statistically significant (*p* = 0.22). This result suggests that subjective cognitive Complaints may not always differentiate between schizophrenia and affective disorders, aligning with findings by Baliga et al. (2020) and Grimstad et al. (2025) that cognitive complaints are influenced by several factors beyond diagnosis.

Our study, like Grimstad et al. (2025), provides some insights into the assessment of subjective cognitive complaints in people with schizophrenia and affective disorders.

Subjectivity and metacognition can be extremely sensitive to the surrounding culture.

In this context, the SSTICS, originally Canadian, has been used in more than 10 different languages and cultures. Using a new measure capable of cultural adaptation (SSTIC-E), we were able to examine the relationship between these self-perceived cognitive difficulties and objective cognitive performance and clinical characteristics. The study highlighted that subjective reports often diverge from objective cognitive outcomes. This discrepancy suggests that subjective cognitive assessments may be influenced more by clinical factors, such as depressive symptoms, than by actual cognitive deficits.

### Socio-demographic and clinical features

Numerous studies, such as those by Zhornitsky et al. (2011) and Sellwood et al. (2013), emphasize the role of mood, awareness, and psychiatric symptoms in shaping subjective cognitive complaints. Additionally, studies like those by Baliga et al. (2020) and Haddad et al. (2021) found links between subjective cognitive complaints and clinical features like depression and social functioning.

Our results confirm some of these trends. We observed that patients with schizophrenia who had higher subjective cognitive complaints (as measured by the SSTIC-E) also reported greater impairments in social functioning, like findings by Baliga et al. (2020).

Moreover, depression was positively linked to subjective cognitive complaints, Aligning with Haddad et al.’s (2021) conclusions that mood disorders exacerbate cognitive self-reports.

### Cross-cultural and transdiagnostic approaches

In another Arabic country, Johnson et al. (2009) validated the SSTICS for Arabic-speaking Tunisian patients, reinforcing the cross-cultural applicability of the scale.

Additionally, studies like those by Potvin et al. (2017) and Chuang et al. (2019) highlighted the importance of considering factors such as internalized stigma and insight into cognitive deficits when assessing subjective cognitive complaints, when our study further extends the cross-cultural validity of the SSTIC-E by adapting it to the Arabic-speaking population different than Tunisian, or Lebanese (Haddad et al., 2021).

### Reconciling differences

Contrasting the results of this study with the literature, the lack of statistical significance in SSTIC-E scores between schizophrenia and affective disorder groups may suggest that both disorders share substantial commonalities in subjective cognitive complaints. This is supported by recent transdiagnostic research, which highlights overlapping cognitive and functional impairments across psychiatric conditions (Grimstad et al., 2025; Millan et al., 2012). Furthermore, cultural and sociodemographic factors unique to this study’s population may influence the way cognitive difficulties are perceived and reported, potentially reducing the observable differences between groups.

### Limitations

Although the study included 210 participants, the sample may not be fully representative of the broader population due to its concentration of Emirati nationals (60.3%). Its cross-sectional design prevents establishing causal links between cognitive complaints, cognitive performance, and psychiatric diagnoses. Influences such as medication, symptom severity, and psychosocial factors were not thoroughly examined, affecting outcomes.

The reliance on self-reported tools like the SSTIC-E may introduce bias, including over- or underreporting of cognitive problems. Self-reported cognitive complaint scales are limited by poor correlation with objective measures, confounding by mood symptoms, response bias, lack of standardization, reduced insight in progressive disease, and inferior predictive value compared to informant reports.

Furthermore, the absence of data from other standardized clinical scales such as the PANSS, Calgary Depression Scale, or HDRS reduces the depth of correlations with clinical assessment, potentially influencing the validity and comprehensiveness of the study’s findings. In addition, we did not use an informant-based cognitive assessment tool, a structured questionnaire, or an interview designed to evaluate a patient’s cognitive function by collecting information from a knowledgeable informant (typically a family member, caregiver, or close acquaintance) rather than directly from the patient.

These tools assess changes in cognition, behavior, and functional abilities over time, relying on the informant’s observations of the patient’s day-to-day functioning and cognitive decline. Informant-based tools have demonstrated reasonable sensitivity and specificity for detecting dementia and cognitive impairment in various settings, including primary care, hospital, and community populations. Informant-based cognitive assessment tools are more valid and have greater predictive value than self-reported scales for early detection of cognitive impairment. They generally outperform self-reported scales in the early detection of cognitive impairment across culturally diverse populations, but their accuracy and validity can be influenced by cultural, linguistic, and informant-related factors. Self-reported scales, such as the SSTICS or the SSTIC-E, while easy to administer, are more vulnerable to cultural, linguistic, and educational influences, and their correlation with objective cognitive performance is weaker than informant-based tools, especially in diverse populations.

## Conclusion

This study is among the first to validate the Arabic version of SSTICS, confirming its reliability and usefulness in Arabic-speaking psychiatric populations, not only focused on schizophrenia. It is in line with the Tunisian and Lebanese studies but is more suited to the regional political union of the Gulf Cooperation Council (GCC), which includes Bahrain, Kuwait, Oman, Qatar, Saudi Arabia, and the United Arab Emirates.

The adaptation of the SSTIC-E expands the availability of culturally appropriate cognitive assessment tools and supports its role as a rapid, self-reported measure to detect subjective cognitive complaints. When used alongside the Montreal Cognitive Assessment (MoCA), which is an objective cognitive complaints measurement tool, the SSTIC-E reveals discrepancies between perceived and measured cognitive deficits and should be administered. With care, considering the cultural differences, especially when we talk about the education level of participants (Homayoun et al., 2011).

This highlights the importance of combining subjective and objective assessments to enhance diagnostic accuracy and inform individualized treatment planning (Allott et al., 2025; Egeland et al., 2003; Grimstad et al., 2025; Homayoun et al., 2011; Lepage et al., 2025). The study also found that women reported more cognitive complaints than men, suggesting gender-based differences in the perception of cognitive dysfunction. This finding supports the need for gender-sensitive approaches in psychiatric assessment and intervention. By focusing on Emirati nationals and incorporating regional factors, the study offers valuable insights into the mental healthcare context of the UAE, emphasizing.

the influence of culture, treatment, or cognitive remediation, and symptom severity on cognitive reporting. The successful validation of the Arabic SSTIC-E contributes significantly to the Understanding of cognitive impairments in psychiatric populations, especially in non-Western contexts.

The results support the integration of the SSTIC-E into routine clinical practice with different mental health conditions and encourage further research into cognitive dysfunction as a transdiagnostic feature across psychiatric conditions. The study also advocates for personalized, culturally informed care and underscores the need for longitudinal and cross-cultural investigations to refine cognitive assessment tools and interventions. Future studies should also incorporate validated psychopathology scales to assess the relationship between symptom severity and subjective cognitive complaints. Longitudinal designs, inclusion of biological markers, and use of informant-based cognitive tools may further clarify how subjective and objective cognition interact across psychiatric disorders.

## Data Availability

The raw data supporting the conclusions of this article will be made available by the authors, without undue reservation.

## References

[ref1] Abramovitch A. Short T. Schweiger A. (2021). The C factor: cognitive dysfunction as a transdiagnostic dimension in psychopathology. Clin. Psychol. Rev. 86:102007. doi: 10.1016/j.cpr.2021.102007, PMID: 33864968

[ref2] Al-Krenawi A. Graham J. R. Al-Bedah E. A. Kadri H. M. Sehwail M. A. (2009). Cross-national comparison of middle eastern university students: help-seeking behaviors, attitudes toward helping professionals, and cultural beliefs about mental health problems. Community Ment. Health J. 45, 26–36. doi: 10.1007/s10597-008-9175-2 19067161

[ref3] Allott K. Bryce S. Douglas K. Stainton A. Wood S. J. Bowie C. R. (2025). Formulating cognitive functioning to guide personalised treatment for people diagnosed with mental disorders. Acta Psychiatr. Scand. 1–15. doi: 10.1111/acps.13811

[ref4] Amro I. Hamadi A. M. A. Salem A. A. E. Chivese T. Wilkins S. S. Khaled S. M. (2025). Population-based norms for the Montreal cognitive assessment in Arab adults. Brain Behav 15:e70287. doi: 10.1002/brb3.70287, PMID: 39924707 PMC11807847

[ref5] Baliga S. P. Kamath R. M. Kedare J. S. (2020). Subjective cognitive complaints and its relation to objective cognitive performance, clinical profile, clinical insight, and social functioning in patients of schizophrenia: a cross-sectional study. Indian J. Psychiatry 62, 178–185. doi: 10.4103/psychiatry.IndianJPsychiatry_639_19, PMID: 32382178 PMC7197831

[ref6] Barch D. M. Karcher N. Moran E. (2022). Reinventing schizophrenia - embracing complexity and complication. Schizophr. Res. 242, 7–11. doi: 10.1016/j.schres.2021.11.037 34893361

[ref7] Bengochea Seco R. Gil Sanz D. Fernández Modamio M. Arrieta Rodríguez M. Sánchez Calleja R. Prat Solís R. . (2010). Cognitive complaints in schizophrenia: relationship with insight and other cognitive measures. Rev. Psiquiatr. Salud Ment. 3, 55–60. doi: 10.1016/j.rpsm.2010 23445930

[ref8] Bora E. Pantelis C. (2016). Social cognition in schizophrenia in comparison to bipolar disorder: a meta-analysis. Schizophr. Res. 175, 72–78. doi: 10.1016/j.schres.2016.04.018 27117677

[ref9] Capitulo K. L. Cornelio M. A. Lenz E. R. (2001). Translating the short version of the perinatal grief scale: process and challenges. Appl. Nurs. Res. 14, 165–170. doi: 10.1053/apnr.2001.22377, PMID: 11481595

[ref10] Chavez-Baldini U. Y. Nieman D. H. Keestra A. Lok A. Mocking R. J. T. de Koning P. . (2023). The relationship between cognitive functioning and psychopathology in patients with psychiatric disorders: a transdiagnostic network analysis. Psychol. Med. 53, 476–485. doi: 10.1017/S0033291721001781, PMID: 34165065 PMC9899564

[ref11] Chelune G. J. Heaton R. K. Lehman R. A. (1986). “Neuropsychological and personality correlates of patients’ complaints of disability” in Advances in clinical neuropsychology. eds. Goldstein G. Tarter R. E. (Boston, MA: Springer US), 95–126.

[ref12] Chuang S. P. Wu J. Y. W. Wang C. S. (2019). Self-perception of mental illness, and subjective and objective cognitive functioning in people with schizophrenia. Neuropsychiatr. Dis. Treat. 15, 967–976. doi: 10.2147/NDT.S193239, PMID: 31118637 PMC6499497

[ref13] Cotter J. Granger K. Backx R. Hobbs M. Looi C. Y. Barnett J. H. (2018). Social cognitive dysfunction as a clinical marker: a systematic review of meta-analyses across (30) clinical conditions. Neurosci. Biobehav. Rev. 84, 92–99. doi: 10.1016/j.neubiorev.2017.11.014, PMID: 29175518

[ref14] Derouesné C. Lacomblez L. Thibault S. Leponcin M. (1999). Memory complaints in young and elderly subjects. Int. J. Geriatr. Psychiatry 14, 291–301. doi: 10.1002/(SICI)1099-1166(199904)14:4<291::AID-GPS902>3.0.CO;2-8 10340191

[ref15] Duffy M. E. (2006). Translating instruments into other languages: basic considerations. Clin. Nurse Spec. 20, 225–226. doi: 10.1097/00002800-200609000-00006 16980789

[ref16] East-Richard C. R-Mercier A. Nadeau D. Cellard C. (2020). Transdiagnostic neurocognitive deficits in psychiatry: a review of meta-analyses. Can. Psychol. 61, 190–214. doi: 10.1037/cap0000196

[ref17] Egeland J. Sundet K. Rund B. R. . (2003). Sensitivity and specificity of memory dysfunction in schizophrenia: a comparison with major depression. J. Clin. Exp. Neuropsychol. 25, 79–93. doi: 10.1076/jcen.25.1.79.13630 12607174

[ref18] Khatib H. E. Alyafei A. Shaikh M. (2023). Understanding experiences of mental health help-seeking in Arab populations around the world: a systematic review and narrative synthesis. BMC Psychiatry 23:324. doi: 10.1186/s12888-023-04827-4 37161342 PMC10170733

[ref19] Elbarazi I. Abdullahi A. S. Abdel Aziz K. Stip E. Elkonaisi I. Fahim F. . (2025). Perceptions about brain health among the United Arab Emirates population using the global brain survey: a cross-sectional study. Front. Public Health 13:1518938. doi: 10.3389/fpubh.2025.1518938, PMID: 40135156 PMC11932854

[ref20] Grimstad K. Sørensen H. Vaskinn A. Mohn C. Olsen S. H. Andreassen O. A. . (2025). Subjective cognition in schizophrenia and bipolar disorder: investigation of group differences and associations with objective cognition and clinical characteristics using a novel measure of subjective cognition. Schizophr. Res. Cogn. 40:100345. doi: 10.1016/j.scog.2025.100345 39989506 PMC11846586

[ref21] Gualtieri C. T. Johnson L. G. (2008). Age-related cognitive decline in patients with mood disorders. Prog. Neuro-Psychopharmacol. Biol. Psychiatry 32, 962–967. doi: 10.1016/j.pnpbp.2007.12.030 18243461

[ref22] Haddad C. Salameh P. Sacre H. Polin C. Clément J. P. Calvet B. (2021). Subjective cognitive complaints and relations to objective cognitive performance among Lebanese patients with schizophrenia. BMC Psychiatry 21:549. doi: 10.1186/s12888-021-03564-w 34753438 PMC8576858

[ref23] Hamid A. A. R. M. Darweesh A. H. M. (2020). Cognitive impairment in psychiatric patients in the United Arab Emirates. Appl. Neuropsychol. Adult 27, 87–93. doi: 10.1080/23279095.2018.1488715, PMID: 30375887

[ref24] Homayoun S. Nadeau-Marcotte F. Luck D. Stip E. (2011). Subjective and objective cognitive dysfunction in schizophrenia–is there a link? Front. Psychol. 2:148. doi: 10.3389/fpsyg.2011.00148, PMID: 21779267 PMC3131547

[ref25] Johnson I. Kebir O. Ben Azouz O. Dellagi L. Rabah Y. Tabbane K. (2009). The self-assessment scale of cognitive complaints in schizophrenia: a validation study in the Tunisian population. BMC Psychiatry 9:66. doi: 10.1186/1471-244X-9-66 19814827 PMC2766383

[ref26] Lau J. H. Abdin E. Vaingankar J. A. Shafie S. Sambasivam R. Shahwan S. . (2021). Confirmatory factor analysis and measurement invariance of the English, mandarin, and Malay versions of the SF- 12v2 within a representative sample of the multi-ethnic Singapore population. Health Qual. Life Outcomes 19:80. doi: 10.1186/s12955-021-01709-9 33691707 PMC7944897

[ref27] Lecardeur L. Briand C. Prouteau A. Lalonde P. Nicole L. Lesage A. . (2009). Preserved awareness of their cognitive deficits in patients with schizophrenia: convergent validity of the SSTICS. Schizophr. Res. 107, 303–306. doi: 10.1016/j.schres.2008 18835134

[ref28] Lecomte Y. Stip E. Caron J. Renaud S. (2007). Une étude exploratoire de l’adaptation de personnes souffrant de schizophrénie. Sante Ment. Que. 32, 137–158. doi: 10.7202/016513ar, PMID: 18253665

[ref29] Lepage M. Bodnar M. Bowie C. R. (2014). Neurocognition: clinical and functional outcomes in schizophrenia. Can. J. Psychiatr. 59, 5–12. doi: 10.1177/070674371405900103 PMC4079224 24444318

[ref30] Lepage M. Guimond S. Raedler T. McNeely H. E. Ungar T. Margolese H. C. . (2025). Strategies for achieving better cognitive health in individuals with schizophrenia spectrum: a focus on the Canadian landscape. Can. J. Psychiatr. 70, 85–89. doi: 10.1177/07067437241261928 PMC11572005 39051555

[ref31] Li C. Hong Y. Yang X. Zeng X. Ocepek‐Welikson K. Eimicke J. P. . (2023). The use of subjective cognitive complaints for detecting mild cognitive impairment in older adults across cultural and linguistic groups: a comparison of the cognitive function instrument to the Montreal cognitive assessment. Alzheimers Dement. 19, 1764–1774. doi: 10.1002/alz.12804 36222321 PMC10090224

[ref32] Lysaker P. H. Minor K. S. Lysaker J. T. Hasson-Ohayon I. Bonfils K. Hochheiser J. . (2020). Metacognitive function and fragmentation in schizophrenia: relationship to cognition, self-experience and developing treatments. Schizophr. Res. Cogn. 19:100142. doi: 10.1016/j.scog.2019.100142, PMID: 31828019 PMC6889776

[ref33] Millan M. J. Agid Y. Brüne M. Bullmore E. T. Carter C. S. Clayton N. S. . (2012). Cognitive dysfunction in psychiatric disorders: characteristics, causes and the quest for improved therapy. Nat. Rev. Drug Discov. 11, 141–168. doi: 10.1038/nrd3628, PMID: 22293568

[ref34] Moritz S. Andreou C. Schneider B. C. Wittekind C. E. Menon M. Balzan R. P. . (2014). Sowing the seeds of doubt: a narrative review on metacognitive training in schizophrenia. Clin. Psychol. Rev. 34, 358–366. doi: 10.1016/j.cpr.2014.04.004, PMID: 24866025

[ref35] Moselhy H. F. Ghubach R. El-Rufaie O. Zoubeidi T. Badrinath P. Sabri S. . (2012). The association of depression and anxiety with unhealthy lifestyle among United Arab Emirates adults. Epidemiol. Psychiatr. Sci. 21, 213–219. doi: 10.1017/S2045796011000709, PMID: 22789171

[ref36] Nasreddine Z. S. Phillips N. A. Bédirian V. Charbonneau S. Whitehead V. Collin I. . (2005). The Montreal cognitive assessment, MoCA: a brief screening tool for mild cognitive impairment. J. Am. Geriatr. Soc. 53, 695–699. doi: 10.1111/j.1532-5415.2005.53221.x, PMID: 15817019

[ref37] Potvin S. Aubin G. Stip E. (2017). Subjective cognition in schizophrenia. Encéphale 43, 15–20. doi: 10.1016/j.encep.2016.01.002 26923995

[ref38] Potvin S. Briand C. Prouteau A. Bouchard R. H. Lipp O. Lalonde P. . (2005). CANTAB explicit memory is less impaired in addicted schizophrenia patients. Brain Cogn. 59, 38–42. doi: 10.1016/j.bandc.2005.04.002 15913868

[ref39] Potvin S. Pelletier J. Stip E. (2014). Neurocognitive insight in schizophrenia: a meta- analysis. Sante Ment. Que. 39, 183–200, PMID: 25590551

[ref40] Prouteau A. Verdoux H. Briand C. Lesage A. Lalonde P. Nicole L. . (2004). Self-assessed cognitive dysfunction and objective performance in outpatients with schizophrenia participating in a rehabilitation program. Schizophr. Res. 69, 85–91. doi: 10.1016/j.schres.2003.08.011, PMID: 15145474

[ref7001] Raffard E. Lebrun C. Bayard S. Macgregor A. Capdevielle D. (2020). Self-awareness deficits of cognitive impairment in individuals with schizophrenia. Really? Front. Psychia. 11:731. 10.3389/fpsyt.2020.00731 PMC7406784 32848912

[ref41] Sellwood W. Morrison A. P. Beck R. Heffernan S. Law H. Bentall R. P. (2013). Subjective cognitive complaints in schizophrenia: relation to antipsychotic medication dose, actual cognitive performance, insight and symptoms. PLoS One 8:e83774. doi: 10.1371/journal.pone.0083774, PMID: 24376745 PMC3869800

[ref42] Sheffield J. M. Kandala S. Tamminga C. A. Pearlson G. D. Keshavan M. S. Sweeney J. A. . (2017). Transdiagnostic associations between functional brain network integrity and cognition. JAMA Psychiatry 74, 605–613. doi: 10.1001/jamapsychiatry.2017.0669, PMID: 28467520 PMC5539843

[ref43] Shin Y. J. Joo Y. H. Kim J. H. (2016). Self-perceived cognitive deficits and their relationship with internalized stigma and quality of life in patients with schizophrenia. Neuropsychiatr. Dis. Treat. 12, 1411–1417. doi: 10.2147/NDT.S108537, PMID: 27366073 PMC4913959

[ref44] Snyder H. R. Miyake A. Hankin B. L. (2015). Advancing understanding of executive function impairments and psychopathology: bridging the gap between clinical and cognitive approaches. Front. Psychol. 6:328. doi: 10.3389/fpsyg.2015.00328 25859234 PMC4374537

[ref45] Stip E. Al Mugaddam F. Abdel Aziz K. Amiri L. Javaid S. F. Arnone D. . (2024a). Cross-cultural differences through subjective cognition: illustration in translatology with the SSTIC-E in the UAE. Front. Psychol. 15:1125990. doi: 10.3389/fpsyg.2024.1125990 38515979 PMC10956416

[ref46] Stip E. Al Mugaddam F. Nauman J. Baki A. A. Potvin S. (2022). Subjective cognitive complaints in first episode psychosis: a focused follow-up on sex effect and alcohol usage. Schizophr. Res. Cogn. 30:100267. doi: 10.1016/j.scog.2022.100267, PMID: 36042936 PMC9420513

[ref47] Stip E. Alkaabi A. A. AlAhbabi M. Al-Mugaddam F. Lungu O. Albastaki M. F. . (2024b). Measuring subjective cognitive complaints with covid-19 brain fog using the subjective scale to investigate cognition (SSTICS). Appl. Neuropsychol. Adult, 1–13. doi: 10.1080/23279095.2024.2422926 39531522

[ref48] Stip E. Caron J. Renaud S. Pampoulova T. Lecomte Y. (2003). Exploring cognitive complaints in schizophrenia: the subjective scale to investigate cognition in schizophrenia. Compr. Psychiatry 44, 331–340. doi: 10.1016/S0010-440X(03)00086-5 12923712

[ref49] Stip E. Caron J. Tousignant M. Lecomte Y. (2017). Suicidal ideation and schizophrenia: contribution of appraisal, stigmatization, and cognition. Can. J. Psychiatr. 62, 726–734. doi: 10.1177/0706743717715207, PMID: 28673099 PMC5638189

[ref50] Stratta P. Pacitti F. Rossi R. Chialastri S. Santarelli V. Marucci C. . (2020). Subjective scale to investigate cognition in schizophrenia (SSTICS): a validation study in Italian population. Riv. Psichiatr. 55, 98–105. doi: 10.1708/3333.33024, PMID: 32202547

[ref51] Zhornitsky S. Potvin S. Aubin G. Stip E. (2011). Relationship between insight into cognition, extrapyramidal symptoms and mental illness in schizophrenia. Aust. N. Z. J. Psychiatry 45, 604–605. doi: 10.3109/00048674.2011.561483, PMID: 21355813

